# A highly efficient indirect ELISA and monoclonal antibody established against African swine fever virus pK205R

**DOI:** 10.3389/fimmu.2022.1103166

**Published:** 2023-01-09

**Authors:** Liwei Li, Sina Qiao, Jiachen Liu, Yanjun Zhou, Wu Tong, Shishan Dong, Changlong Liu, Yifeng Jiang, Ziqiang Guo, Haihong Zheng, Ran Zhao, Guangzhi Tong, Guoxin Li, Fei Gao

**Affiliations:** ^1^ Shanghai Veterinary Research Institute, Chinese Academy of Agricultural Sciences, Shanghai, China; ^2^ College of Veterinary Medicine, Hebei Agricultural University, Baoding, China; ^3^ Xiamen Center for Animal Disease Control and Prevention, Xiamen, China; ^4^ Jiangsu Co-Innovation Center for the Prevention and Control of Important Animal Infectious Disease and Zoonose, Yangzhou University, Yangzhou, China

**Keywords:** ASFV pK205R, 293i cells, epitope, indirect ELISA, recombinant PRRSV

## Abstract

African swine fever (ASF) is a contagious infectious disease with high lethality which continuously threatens the global pig industry causing huge economic losses. Currently, there are no commercially available vaccines or antiviral drugs that can effectively control ASF. The pathogen of ASF, ASF virus (ASFV) is a double-stranded DNA virus with a genome ranging from 170 to 193 kb and 151 to 167 open reading frames in various strains, which encodes 150–200 proteins. An effective method of monitoring ASFV antibodies, and specific antibodies against ASFV to promote the development of prevention techniques are urgently needed. In the present study, pK205R of ASFV was successfully expressed in mammalian cells using a suspension culture system. An indirect enzyme-linked immunosorbent assay (ELISA) based on the purified pK205R was established and optimized. The monoclonal antibody (mAb) against pK205R recognized a conservative linear epitope (^2^VEPREQFFQDLLSAV^16^) and exhibited specific reactivity, which was conducive to the identification of the recombinant porcine reproductive and respiratory syndrome virus (PRRSV) expressing pK205R. The ELISA method efficiently detected clinical ASFV infection and revealed good application prospects in monitoring the antibody level *in vivo* for recombinant PRRSV live vector virus expressing the ASFV antigen protein. The determination of the conserved linear epitope of pK205R would contribute to further research on the structural biology and function of pK205R. The indirect ELISA method and mAb against ASFV pK205R revealed efficient detection and promising application prospects, making them ideal for epidemiological surveillance and vaccine research on ASF.

## 1 Introduction

African swine fever (ASF) is an acute and highly contagious disease caused by the African swine fever virus (ASFV), which is characterized by high fever, internal organ bleeding, and other clinical symptoms (1). This disease was first discovered in Kenya in 1921. An outbreak occurred in Georgia in 2007 and quickly spread to Russia and the Caucasus (2). Currently, no effective vaccine or drug is available against this disease; therefore, strict biosafety measures are the most effective way to prevent and control ASF (3, 4). ASF was first reported in August 2018 in China, and the emergence and prevalence of naturally occurring less virulent and naturally gene-deleted ASFV strains in domestic pigs have been identified in recent years (5–9).

ASF vaccination approaches include inactivated vaccines, live attenuated vaccines (LAVs), subunit, DNA, and virus-vectored vaccines (10, 11). Several gene-deleted ASFV vaccine candidates have been reported in many countries (12–16). Thus, LAVs pose a slight risk; as in rare cases, attenuated strains may regain pathogenicity, causing the spread of the disease; they have the potential to cause post-vaccination reactions and side effects. The structural proteins p54, p30, p72, and hemagglutinin CD2v have traditionally been the main targets of subunit and DNA vaccines. More recent studies using antigen cocktails of up to 47 different ASFV genes delivered by adenovirus, alphavirus, and vaccinia virus vectors demonstrated the induction of strong antigen-specific cellular responses (3).

ASFV contains a linear double-stranded 170–193 kb DNA genome, encoding more than 150 proteins, although the functions of more than half of these proteins remain to be clarified (17). Among these, p12 (*O61R*), p17 (*D117L*), p30 (*CP204L*), p54 (*E183L*), and p72 (*B646L*) are identified to be involved in viral replication, immunological evasion, and transmission of pathogens. A previous study detected 12 viral proteins as the main targets of serological immunity (18). Among them, pK205R was identified as a potentially useful serological diagnostic antigen for the detection of IgM responses 11 days post-infection with ASFV (19). ORF *K205R* encodes pK205R, an early viral protein that contains a conserved amino acid sequence and is expressed in the early stages of infection from 4 h post-infection (hpi). Prokaryotic pK205R has high antigenicity and can be recognized by hyperimmune antisera from infected pigs in enzyme-linked immunosorbent assay (ELISA) testing, suggesting that pK205R has the potential to be used for the detection of ASFV-specific antibodies (18, 20, 21). Consistently high serological responses against pK205R were observed at late stages of infection compared with the response to structural proteins in domestic pigs and bushpigs infected with a range of different viral isolates. This suggests that ELISA against pK205R may be useful in distinguishing animals persistently infected with viruses from animals immunized with vaccines incorporating viral structural proteins (20). Adenovirus-vectored pK205R elicits robust immune responses in swine (22).

Mammalian cells are frequently and widely used for the stable expression and purification of mAb, interferons, growth factors, and viral antigens (23). Mammalian cell systems have more effective and precise post-translational modifications than that in the prokaryotic system; moreover, they express recombinant proteins that are very close to natural proteins in terms of molecular structure, physicochemical properties, and biological functions. Using a serum-free and composition-simple medium for the suspension culture of mammalian cells contributes to massive purification and production and also increases the expression level of recombinant proteins. Herein, we expressed recombinant pK205R in mammalian cells using the suspension culture system and established an indirect ELISA and mAb against pK205R, which revealed efficient detection and promising application perspectives.

## 2 Materials and methods

### 2.1 Cells, viruses, and sera

Various cell cultures, including 293T (ATCC, Manassas, VA, USA), SP2/0 (ATCC), MARC-145 (ATCC), porcine alveolar macrophages (PAMs), and suspension-cultured 293i cells (Thermo Fisher Scientific, Waltham, MA, USA) were maintained in our laboratory. A full-length cDNA clone of the HP-PRRSV attenuated strain vHuN4-F112 obtained by serial cell passage (GenBank accession no. EF635006) was used as the backbone for the insertion of ASFV *K205R* from SY18 (GenBank accession no. MH766894.1). The resultant recombinant virus was compared with the parental virus, vHuN4-F112. PRRSV titers in MARC-145 cells were determined using the standard median tissue culture infective dose (TCID_50_) following the Reed and Muench method (24). Swine serum samples of a virulent ASFV strain (wild-type ASFV) were stored until further use. Swine serum samples (n = 162) were collected from pig farms. Furthermore, serum samples positive for classical swine fever virus (CSFV), PRRSV, foot and mouth disease virus (FMDV), type 2 porcine circovirus (PCV2), porcine epidemic diarrhea virus, porcine circovirus (PEDV), and pseudorabies virus (PRV) were conserved in our laboratory.

### 2.2 Expression and purification of recombinant pK205R

Based on the *K205R* gene sequence of the ASFV SY18 strain, specific primers were designed using Oligo7 software, and pcDNA3.4-*K205R*-strep plasmid was constructed using the pcDNA3.4 TOPO™ TA Kit (Thermo Fisher Scientific). The recombinant eukaryotic expression vector pcDNA3.4-*K205R*-strep was transiently transfected into 293i cells and maintained in a shaking incubator at 37°C and 8% CO_2_ at 125r/min. The cells were supplemented with enhancers and auxiliaries after 18-22 hours, and incubated in a shaking incubator for 5 days. Cells were identified 1, 3, and 5 days post-transfection (dpt). Cells were collected at 5 dpt, and the recombinant pK205R was purified using StrepTrap beads according to the instructions provided by the manufacturer (General Electric Company, Boston, MA, USA). The collected samples were identified by SDS-PAGE and Western blotting (WB) using an anti-Strep tag antibody (1:4000, ab180957, Abcam, Cambridge, MA, USA).

### 2.3 Establishment of an indirect ELISA against pK205R

The indirect ELISA was optimized using the square titration method. The ELISA plates were first coated with different concentrations of pK205R (100, 200, 400, 800, and 1000 ng/well), and ASFV positive and negative sera were diluted from 1:50 to 1:400 to determine the optimal concentration of protein coating and serum dilution, respectively. The coating solution (carbonate or phosphate), sealing solution (5% skim milk or 5% BSA), and sealing time (37°C for 1h or 37°C for 2h) were optimized. The coating temperature (37°C for 1 h, 37°C for 2 h, 37°C for 4 h, or 4°C overnight) and secondary antibody dilution (6 ×10^3^, 1 × 10^4^, 2 × 10^4^, or 4 × 10^4^) were then optimized. Then, the optimal duration (20, 30, 45, or 60 min) of the serum and secondary antibodies was identified according to the conditions determined above. The color development conditions were optimized using TMB (5, 10, or 15 min at room temperature or 37°C, respectively).

### 2.4 Generation and screening of the mAb against pK205R

Five 4-week-old female BALB/c mice were immunized with purified pK205R *via* intramuscular, subcutaneous, and intraperitoneal multipoint injections. The purified protein (50µg) was mixed with MnJ(β) colloidal manganese adjuvant. A second immunization was performed 10 days after the first one, followed by a third and booster immunization in 7 days gaps using the same method and dose. The immune response of the mice was tested by indirect ELISA, and the spleen cells of mice and SP2/0 cells were hybridized to produce hybridoma cells that secreted specific antibodies against pK205R. Hybridoma cells were screened by indirect ELISA, and wells with high positive values were subcloned three times using the limited dilution method. Monoclonal cells that stably secreted the specific antibody against pK205R were finally selected for expanded culture and preparation of ascites. The ascites was named 2H11, purified using a Pierce™ antibody clean-up kit (Thermo Fisher Scientific), and stored at -30°C.

### 2.5 Specific detection and subtype identification of the mAb against pK205R

To verify the specificity of 2H11, 293T cells were transfected using pCDNA3.4, and pCDNA3.4-*K205R*-strep plasmids for indirect immunofluorescence assay (IFA) and WB analysis using mAb against pK205R (2H11) or anti-strep tag antibody mentioned above. The fluorescence was visualized using an inverted fluorescence microscope (Olympus Corporation, Tokyo, Japan). The subtypes of 2H11 were identified using a monoclonal antibody isotype identification kit (Proteintech Group, Inc., Chicago, USA) according to the instructions provided by the manufacturer. The purified 2H11 was then diluted multiplicatively (1:1000 to 1:1,024,000), and the antibody titer was measured using the indirect ELISA method established above.

### 2.6 Mapping the B-cell epitope

The minimal epitope recognized by the mAb was identified by WB and indirect ELISA. A truncated fragment of the *K205R* gene was ligated into the prokaryotic expression vector pCold-TF and expressed in BL21(DE3) using IPTG (1mM). The truncated protein recognized by the mAb was verified by WB. Based on these results, the pK205R mutant was further truncated. Finally, the peptides were synthesized and coated onto the ELISA plates. The OD_450nm_ value of each short peptide recognized by the mAb was determined by indirect ELISA, and the smallest B-epitope was determined.

### 2.7 Virus rescue and *in vitro* analysis of virological characteristics

The recombinant full-length cDNA clones (pA-K205R harboring the ASFV *K205R* gene) were successfully assembled using the same strategies as previously described (25). The parental plasmid pHuN4-F112 and recombinant plasmid pA-K205R were linearized with *Swa* I, which was immediately downstream of the poly (A) tail, and then gel-purified using the QIAgen PCR purification kit (QIAgen, Hilden, Germany). Subsequently, linearized templates were then subjected to *in vitro* transcription using the T7 mMESSAGE mMachine kit (Ambion-Thermo Fisher Scientific, Waltham, MA, USA). MARC-145 cells were transfected with 2 μg of *in vitro* transcripts and 2 μL of DMRIE-C (Invitrogen). Cells were monitored daily for cytopathic effects (CPEs). Viral supernatants were collected when 80% of cells developed CPEs. The rescued viruses (P1) were passaged 20 times in MARC-145 cells by plaque plaque purification. Viruses were harvested, designated as vA-ASFV-K205R, and stored at -80°C until use. The viral plaque assay and multistep growth curves were performed as previously described (25). The copy levels of *K205R* gene were detected in the recombinant PRRSVs (P5, P10, P15, and P20 viral stocks) by the real-time reverse transcription quantitative PCR (RT-qPCR) detection method as previously described (26).

### 2.8 Application of the indirect ELISA against ASFV pK205R

#### 2.8.1 Coincidence rate experiments of clinical samples

Swine serum samples (n = 162) were analyzed using a commercial ELISA kit (ASFS-5P, Innovative Diagnostics, Grabels, France) and the established ELISA method with optimized conditions to determine the effectiveness and coincidence.

#### 2.8.2 Specificity testing

Serum samples of CSFV, PRV, PRRSV, PEDV, FMDV, PCV2, and ASFV were tested to determine the specificity of the ELISA method.

#### 2.8.3 Sensitivity testing

The ASFV-positive sera were diluted at six dilutions of 1:40, 1:80, 1:160, 1:320, 1:640, and 1:1280 and determined by the ELISA method under optimized conditions to evaluate the sensitivity.

#### 2.8.4 Detection of specific humoral immunity after vA-ASFV-K205R vaccination

For immune efficacy experiments, fifteen 30-day-old PRRSV- and ASFV-free piglets were selected and divided into three groups to evaluate the specific humoral immunity induced by vA-ASFV-K205R. Each group contained five piglets that were fed separately. A 2 mL dose of vA-ASFV-K205R or vHuN4-F112 (dosage:10^5.0^ TCID_50_) was used to vaccinate each piglet (PRRSV S/P < 0.4; n = 5) through intramuscular cervical injections. Each piglet in the mock group was injected with 2 mL of DMEM. The PRRSV-specific antibody titers in the serum samples collected at specified time points were tested using commercial ELISA kits (IDEXX Laboratories, Westbrook, No. 06-40959-04), as previously described. The ASFV K205R-specific antibody titers in the serum samples collected at the specified time points were tested using the indirect ELISA established above. Based on these values, the humoral immunity levels were plotted using GraphPad Prism 6.0.

### 2.9 Statistical analysis

All experiments included at least three independent repeats. Statistical significance was analyzed using the t-test. Statistical significance was considered at *P* < 0.05.

## 3 Results

### 3.1 The recombinant pK205R was obtained successfully from suspension cultured 293i cells

**Figure 1:**
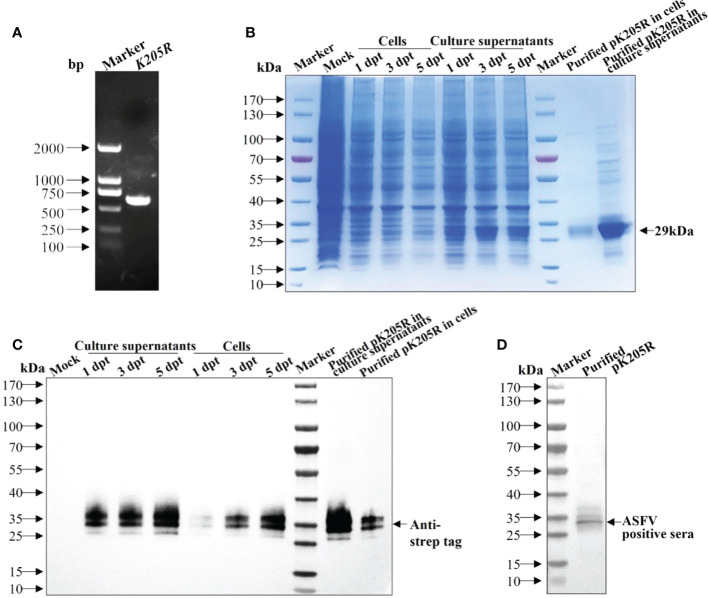
The recombinant pK205R was successfully expressed in 293i cells. **(A)** PCR amplification of *K205R* gene. **(B, C)** Identification of pK205R expression and purification in the culture supernatants and ultrasonic cells by SDS-PAGE **(B)** and WB **(C)**. **(D)** Identification of purified pK205R by WB using an ASFV-positive serum as primary antibody.

The gene fragment of K205R was amplified (**Figure 1A**) and verified using sequencing and was consistent with ASFV SY18 strain. SDS-PAGE showed that pK205Rwas expressed successfully and was mainly secreted into the culture medium. Expression levels gradually increased with increasing transfection time. The expression levels peaked at 5 dpt (**Figure 1B**), which was confirmed by WB results (**Figure 1C**). Using an anti-strep tag antibody forming clear bands of approximately 29 kDa, the purified protein was specifically detected in both the culture medium and ultrasonic supernatant of cells, which was consistent with expectations (**Figure 1C**). Using an ASFV-positive serum as primary antibody, the purified protein was also specifically detected (**Figure 1D**). These results indicated that the recombinant pK205R could be successfully obtained from suspension-cultured 293i cells and specifically recognized by ASFV-positive serum as a suitable antigen for subsequent tests.

### 3.2 The indirect ELISA against pK205R was established and optimized

The obtained pK205R was used as the antigen in indirect ELISA. Using the square titration method, the conditions were optimized as follows. The optimal concentration of pK205R was 100 ng/well. The optimal serum dilution of ASFV-positive and ASFV-negative sera was 1:200. The coating consisted of a carbonate solution at 4°C overnight. The sealing solution consisted of 5% skim milk at 37°C for 2h. The secondary antibody dilution was 1:6000. Then, the optimal durations of serum and secondary antibodies were 30 min and 30 min, respectively. The color development conditions for TMB were 15 min at 37°C. All raw data from the optimization assays will be made available upon request.

### 3.3 The anti-pK205R mAb (2H11) exhibited specific reactivity

**Figure 2:**
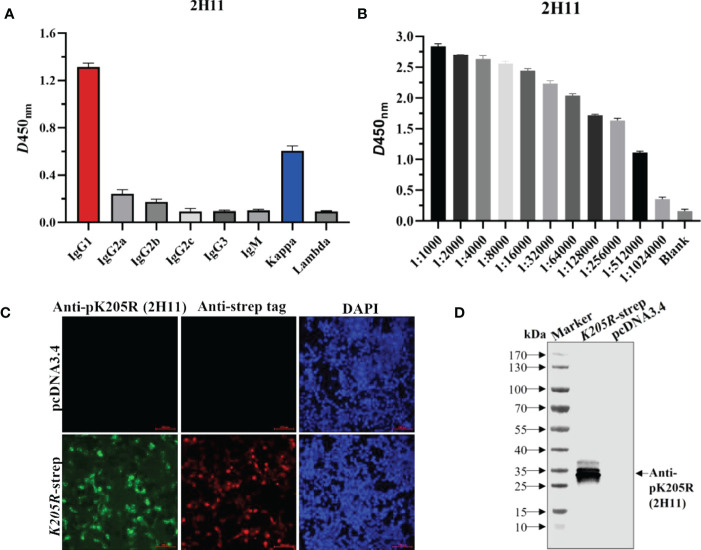
The anti-pK205R mAb (2H11) specifically recognized 293i suspension cells transiently transfected with *K205R*-expressing plasmid. **(A)** Identification of the mAb subtypes and. **(B)** Identification of antibody titer of the purified 2H11 by the ELISA method. **(C)** 293T cells were transfected pcDNA3.4-*K205R*. Cells were fixed at 24 h post-transfection and immunostained with 2H11 and FITC-conjugated goat anti-mouse IgG. Cellular nuclei were counterstained with 1 μg/ml of 4′,6′-diamidino-2-phenylindole (DAPI). **(D)** WB assay was conducted as treated in **(C)** to show the reactivity of 2H11.

Using the above method, the supernatant of hybridoma cells after cloning four times by limiting dilution was determined to be positive, and the positive cells were injected into pristane-treated BALB/c mice to obtain abundant ascetic fluid. The mAb subtypes were identified as IgG1 for the heavy chain and kappa chain for the light chain (**Figure 2A**). The collected ascites was then purified and named 2H11. The detected antibody titer of 2H11 was 1:512,000 as per ELISA (**Figure 2B**). IFA showed that 2H11 and anti-strep tag antibody specifically recognized the expressed-pK205R in 293T cells with green and red fluorescence, respectively, whereas the control did not show any fluorescence (**Figure 2C**). WB showed that 2H11 recognized a specific and clear band with a molecular weight of approximately 29 kDa, whereas it did not react with the control (**Figure 2D**), indicating that the mAb exhibited specific reactivity against pK205R.

### 3.4 2H11 recognized specific linear B-cell epitope

**Figure 3:**
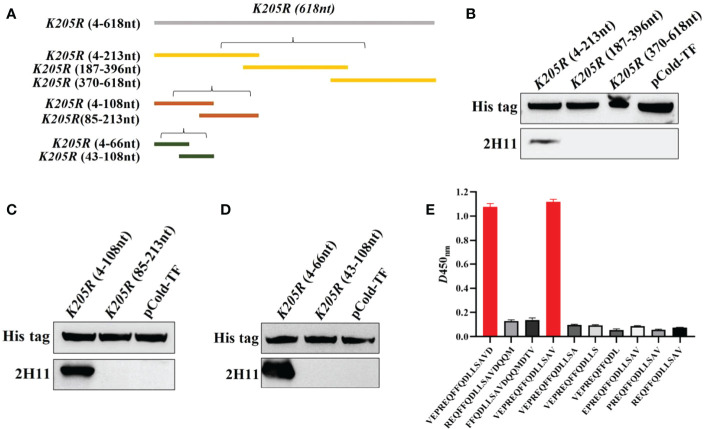
2H11 recognized specific linear B-cell epitope ^2^VEPREQFFQDLLSAV^16^. **(A)** Schematic diagram of *K205R* gene truncated fragments. **(B-D)** A series of truncated pK205R were constructed to pCold-TF and successfully expressed in *E coli* BL21 (DE3) cells. 2H11 was used to detect the truncated pK205R by WB using anti-His tag antibody or 2H11 as primary antibody, respectively. **(E)** Ten different truncated peptides were synthesized and tested by ELISA to show the minimum epitope recognized by 2H11.

The *K205R* gene (1-618nt) was first divided into three segments (P1:4-213nt, P2:187-396nt, P3:370-618nt) and the expression plasmids were constructed (**Figure 3A**). WB showed that 2H11 recognized the P1 region only (**Figure 3B**). Further, the P1 fragment was truncated into two segments (P1-1:4-108nt; P1-2:85-213nt) and 2H11 only recognized the P1-1 region (**Figure 3C**). The P1-1 fragment was then further truncated and divided into two segments (P1-1-1:4-66nt, P1-1-2:43-108nt) and 2H11 recognized the P1-1-1 region only (**Figure 3D**), indicating the epitope located within the P1-1-1 region. Primers used in the experiments are listed in **Table 1**.

**Table 1:** Primers for the truncated protein of pK205R.

Name	Sequences (5′-3′)
*K205R*-S-F	ATGGTTGAGCCACGCGAA
*K205R*-S-R	TTATTTTTCGAACTGCGGGTGGCTCCACTTCTTCATCATCTCTTTGA
pTF-4-213nt-F	atcgaaggtaggcatatgGTTGAGCCACGCGAACAG
pTF-4-213nt-R	ctgcaggtcgacaagcttTTACATAAGGTTGGCCGCCAT
pTF-187-396nt-F	atcgaaggtaggcatatgGAAGAAATGGCGGCCAACCTT
pTF-187-396nt-R	ctgcaggtcgacaagcttTTAAGGTGTTTCACTTGTTTTGCC
pTF-370-618nt-F	atcgaaggtaggcatatgGCCTCAGGCAAAACAAGT
pTF-370-618nt-R	ctgcaggtcgacaagcttTTACTTCTTCATCATCTCTTTGAC
pTF-4-108nt-F	atcgaaggtaggcatatgGTTGAGCCACGCGAACAG
pTF-4-108nt-R	ctgcaggtcgacaagcttTTAAGATGTTTTTTCTTTCATGAT
pTF-85-213nt-F	atcgaaggtaggcatatgGACATCATGAAAGAAAAA
pTF-85-213nt-R	ctgcaggtcgacaagcttTTAGGTCATAAGGTTGGCCGC
pTF-4-66nt-F	atcgaaggtaggcatatgGTTGAGCCACGCGAACAGTTT
pTF-4-66nt-R	ctgcaggtcgacaagcttTTATACAGTGTCCATTTGTTGATCC
pTF-43-108nt-F	atcgaaggtaggcatatgGTGGATCAACAAATGGAC
pTF-43-108nt-R	ctgcaggtcgacaagcttTTAAGATGTTTTTTCTTTCATGATG

For further precise identification of the epitope, ten different truncated peptides were synthesized (**Table 2**) and used to coat the ELISA plates. The results showed that 2H11 recognized the minimum epitope located at amino acids 2–16 of pK205R, with the sequence ^2^VEPREQFFQDLLSAV^16^ (**Figure 3E**).

**Table 2:** Sequences of peptides synthesized in this study.

Name	Peptide Sequences
F1	VEPREQFFQDLLSAVD
F2	REQFFQDLLSAVDQQM
F3	FFQDLLSAVDQQMDTV
F4	VEPREQFFQDLLSAV
F5	VEPREQFFQDLLSA
F6	VEPREQFFQDLLS
F7	VEPREQFFQDL
F8	EPREQFFQDLLSAV
F9	PREQFFQDLLSAV
F10	REQFFQDLLSAV

### 3.5 The epitope recognized by 2H11 was conservative among different ASFV strains

**Figure 4:**
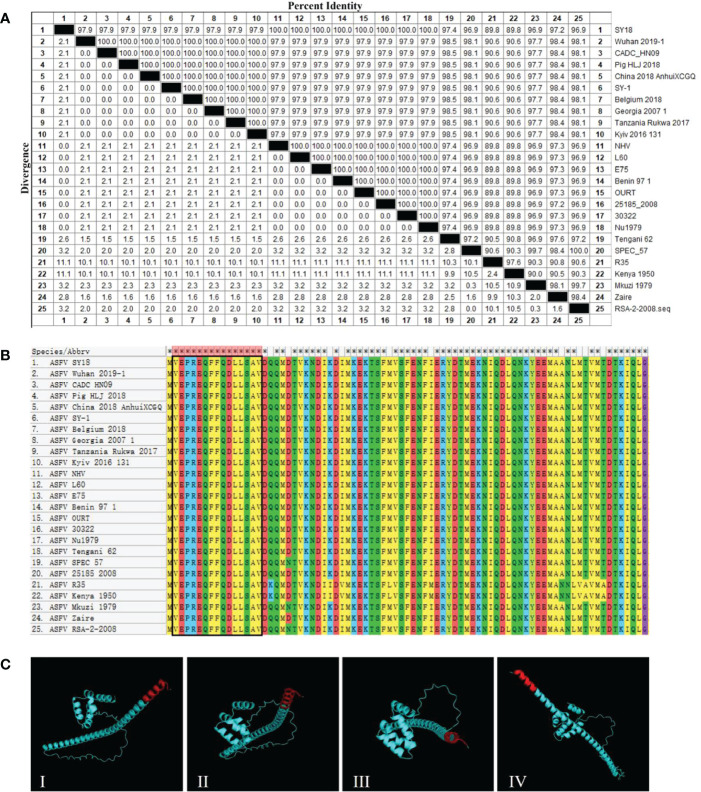
2H11 recognized the conserved epitope of ASFVpK205R. **(A)** The percent identity of pK205R among the 25 representative ASFV strains was analyzed using MEGA. **(B)** Alignment analysis of the epitope (^2^VEPREQFFQDLLSAV^16^) in 25 representative ASFV strains. **(C)** Prediction of the pK205R structure using PyMOL. The epitope recognized by 2H11 is displayed in red color.

To analyze the conservation of the epitope sequence, 25 representative ASFV strains were selected and aligned using MEGA (**Table 3**). The percent identity of pK205R, found in strains with genotype I and II, was higher than 97.9%, but slightly lower than that detected in the other genotypes (**Figure 4A**). Alignment of pK205R sequences showed that the epitope (^2^VEPREQFFQDLLSAV^16^) in all representative strains was conserved (**Figure 4B**). The prediction of the pK205R structure by PyMOL revealed that the epitope (^2^VEPREQFFQDLLSAV^16^) was located at the N-terminal of pK205R (red) and exposed to the surface of the molecule (**Figure 4C**), indicating a conserved epitope of pK205R.

**Table 3:** Reference strains in this study.

NO.	Isolate	Country	Year	Accession no.	Genotype
**1**	ASFV SY18	China	2021	MH766894.1	II
**2**	ASFV Wuhan 2019-1	China	2019	MN393476.1	II
**3**	ASFV CADC_HN09	China	2019	MZ614662.1	II
**4**	ASFV Pig/HLJ/2018	China	2018	MK333180	II
**5**	ASFV China/2018/AnhuiXCGQ	China	2018	MK128995.1	II
**6**	ASFV SY-1	China	2022	OM161110.1	II
**7**	ASFV Belgium 2018	Belgium	2018	LR536725.1	II
**8**	ASFV Georgia 2007/1	Georgia	2007	FR682468.2	II
**9**	ASFV Tanzania/Rukwa/2017	Tanzania	2017	LR813622.1	II
**10**	ASFV Kyiv/2016/131	Ukraine	2016	MN194591	II
**11**	ASFV NHV	Portugal	1968	NC_044943.1	I
**12**	ASFV LH60	Portugal	1960	NC_044941	I
**13**	ASFV E75	Spain	1975	NC_044958	I
**14**	ASFV Benin 97/1	Benin	1997	NC_044956	I
**15**	ASFV OURT	Portugal	1988	NC_044957	I
**16**	ASFV 25185_2008	Italy	2008	MW788410.1	I
**17**	ASFV30322	Italy	2013	MW736600.1	I
**18**	ASFV Nu1979	Italy	1979	MW723481.1	I
**19**	ASFV Tengani 62	Malawi	1962	AY261364	I/V
**20**	ASFV SPEC_57	South Africa	1985	MN394630.3	VIII
**21**	ASFV R35	Uganda	2018	MH025920.1	IX
**22**	ASFV Kenya 1950	Kenya	1950	NC_044944	X
**23**	ASFV Mkuzi 1979	South Africa	1979	NC_044953.1	XII
**24**	ASFV Zaire	Zaire	1977	MN630494	XX
**25**	ASFV RSA_2_2008	South Africa	2008	MN336500	XXII

### 3.6 2H11 specifically recognized the recombinant PRRSV expressing pK205R

**Figure 5:**
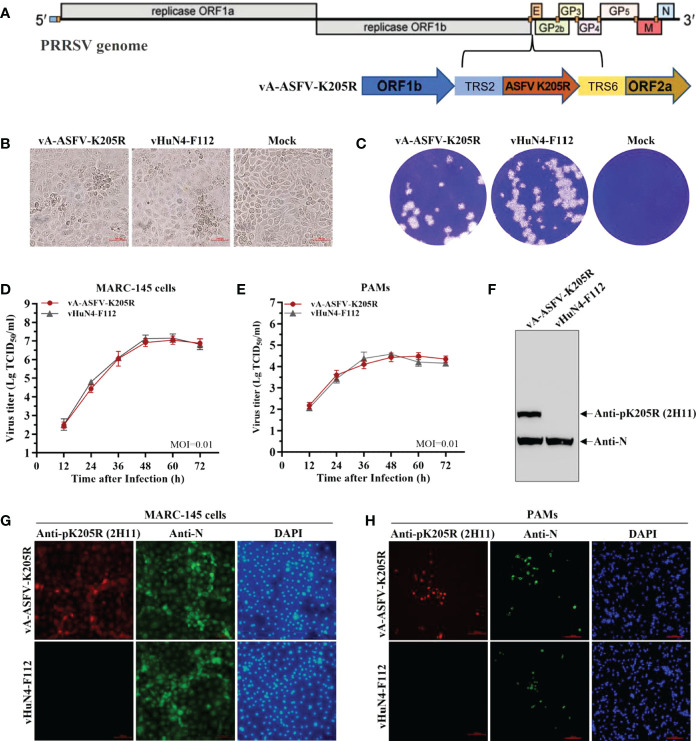
2H11 specifically recognized the recombinant PRRSV expressing pK205R. **(A)** The schematic representation of recombinant PRRSV virus expressing ASFV pK205R construction. **(B, C)** CPE and plaque morphology investigation. MARC-145 cells were infected with vA-ASFV-K205R and vHuN4-F112 (MOI = 0.001). The mock control represented non-infected MARC-145 cells. The MARC-145 cells were monitored or stained with crystal violet at 3 days post-infection. **(D, E)** Growth characteristics of vA-ASFV-K205R and vHuN4-F112 were evaluated in MARC-145 cells **(D)** and PAMs **(E)**. **(F)** MARC-145 cells were infected by vA-ASFV-K205R and vHuN4-F112. WB analysis of cell lysates using an anti-N and anti-pK205R antibody. **(G, H)** IFA against the N protein or pK205R in MARC-145 cells **(G)** and PAMs **(H)** at 36 hpi with vA-ASFV-K205R and vHuN4-F112 (MOI = 0.1). Scale bar = 100 μm.

The recombinant PRRSV virus expressing ASFV pK205R was constructed as shown in **Figure 5A**. To evaluate *in vitro* growth characteristics of vA-ASFV-K205R and vHuN4-F112, CPE, plaque morphology, and virus titers were monitored, as described previously (25). The results showed that the CPEs of vA-ASFV-K205R developed simultaneously with those of the parental strain, vHuN4-F112 (**Figure 5B**); vA-ASFV-K205R and vHuN4-F112 appeared similar in respect to plaque morphology and viral growth analysis (**Figures 5C-E**). We evaluated foreign gene expression of vA-ASFV-K205R. WB showed that 2H11 specifically recognized the vA-ASFV-K205R producing a specific band and did not react with the vHuN4-F112 parental virus. As a control, a laboratory-preserved antibody against the PRRSV N protein was used as the primary antibody, which reacted with both viruses to produce specific bands (**Figure 5F**). MARC-145 cells were infected and tested for IFA by using the prepared 2H11 antibody as the primary antibody. The results showed that 2H11 specifically bound to vA-ASFV-K205R-infected MARC-145 cells, producing red fluorescence, whereas it reacted with vHuN4-F112-infected MARC-145 cells, producing no fluorescence. As a control, the PRRSV N protein antibody was used as the primary antibody and reacted with both viruses to produce a specific green fluorescence (**Figure 5G**). PAMs were used to analyze the expression of pK205R in the target cells of PRRSV infection. The results showed that 2H11 specifically bound to vA-ASFV-K205R-infected PAMs, producing red fluorescence (**Figure 5H**).

**Figure 6:**
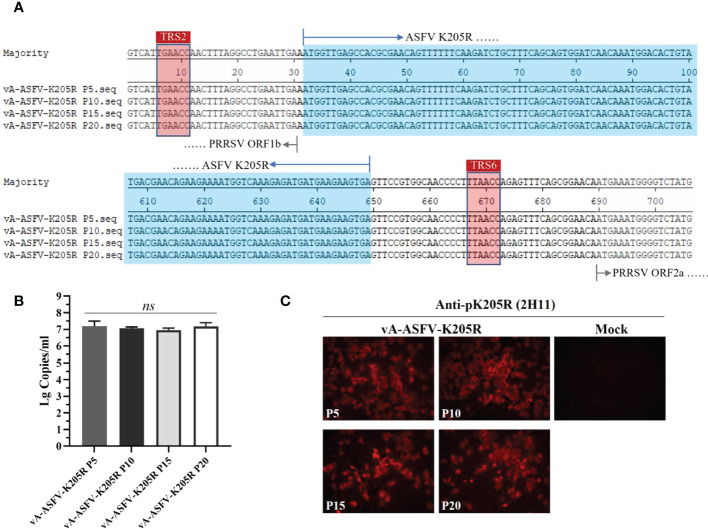
MARC-145 cells were infected by the recombinant virus vA-ASFV-K205R (P5, P10, P15, and P20 viral stocks). Cell culture supernatants were collected at 48 h post infection. **(A)** Sequence alignments to show the genetic stability of ASFV *K205R* gene (P5, P10, P15, and P20 viral stocks). The sequences of foreign gene were displayed in blue color. **(B)** The copy levels of K205R gene were detected in the recombinant PRRSVs (P5, P10, P15, and P20 viral stocks) by RT-qPCR. Data are presented as the mean ± standard deviation of three independent experiments. Statistical significance was analyzed using *t*-tests. *ns*, not significant. **(C)** The pK205R expression in the recombinant PRRSVs (P5, P10, P15 and P20 viral stocks) was detected using 2H11 by IFA.

By the serial cell passage of recombinant virus *in vitro*, the genetic stability of ASFV *K205R* gene (P5, P10, P15, and P20 viral stocks) was detected. Sequence alignment data showed that no nucleic acid mutation was present throughout the full-length of foreign gene, indicating that vA-ASFV-K205R was stable for at least 20 cell passages (**Figure 6A**). The results of RT-qPCR showed that ASFV *K205R* gene could be stably detected in the recombinant PRRSVs (**Figure 6B**). IFA for vA-ASFV-K205R (P5, P10, P15 and P20 viral stocks) demonstrated that 2H11 specifically bound to vA-ASFV-K205R-infected MARC-145 cells producing red fluorescence, indicating that pK205R could be stably expressed in recombinant PRRSVs (**Figure 6C**), which was consistent with the results in **Figure 6B**. Results above indicated that the anti-pK205R mAb generated in this study exhibited a specific response and could be used for the identification and detection of the recombinant virus expressing ASFV pK205R.

### 3.7 The ELISA method revealed efficient detection and application prospects

**Figure 7:**
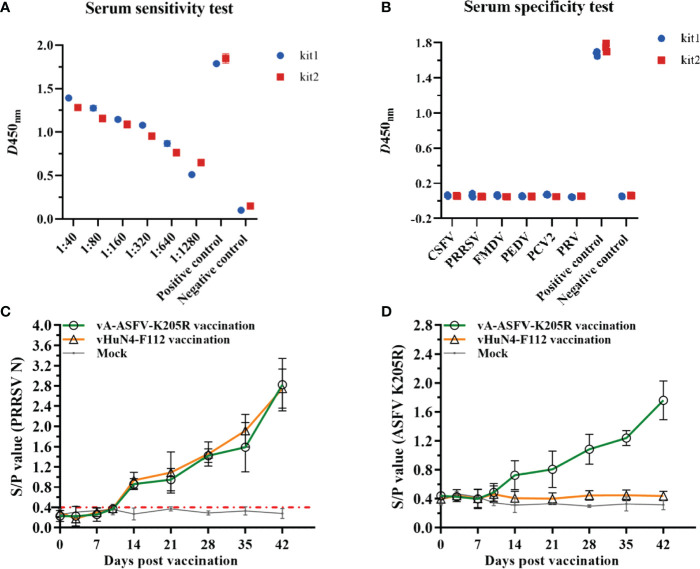
The indirect ELISA method against pK205R efficiently detected clinical samples and recombinant PRRSV virus expressing ASFV antigen. **(A)** Sensitivity testing of the ELISA method and the commercial ELISA kit using positive sera of ASFV. **(B)** Specificity testing of the method using CSFV, PRV, PRRSV, PEDV, FMDV, PCV2 and ASFV sera samples. **(C)** The PRRSV-specific humoral immune response was assessed by the S/P value identified from serum samples collected at the indicated time points from piglets in vA-ASFV-K205R, vHuN4-F112, and mock groups. **(D)** pK205R-specific serum antibodies of the three groups were tested using the indirect ELISA method.

Positive sera for ASFV at different dilutions were detected, and the results showed that the sera appeared positive at 1:1280 (**Figure 7A**), indicating the high sensitivity of the method. Serum samples of CSFV, PRV, PRRSV, PEDV, FMDV, PCV2, and ASFV were tested using the established ELISA method. The S/N values of the standard ASFV-positive sera were significantly greater than 1.6, while the S/N values of the remaining sera were all less than 0.2, which met the criteria for negative sera, indicating good specificity of the method. The established ELISA method was performed using commercial kits (**Figure 7B**). Furthermore, 54 of 162 clinical serum samples tested positive by the established ELISA method, while 53 samples tested positive by the commercial kit (**Table 4**), indicating that the established ELISA method is suitable for the diagnosis of clinical samples. Finally, ELISA was used to monitor the antibody levels *in vivo* of recombinant PRRSV virus expressing ASFV pK205R. The groups vaccinated with vA-ASFV-K205R or vHuN4-F112 produced PRRSV-specific immune effects. All piglets showed seroconversion by 14 days post vaccination (dpv), and the average S/P showed a peak of approximately 2.8 at 42 dpv (**Figure 7C**), demonstrating that vA-ASFV-K205R vaccination induced high levels of PRRSV-specific antibodies. Whereas, the ASFV-specific antibody levels began to increase at 14 dpv in piglets vaccinated with vA-ASFV-K205R and peaked at 42 dpv (**Figure 7D**). During the study period, PRRSV-specific and ASFV-specific antibodies in the mock group piglets remained negative (**Figures 6C, D**). In summary, the established ELISA method has great potential for the specific and effective detection of clinical ASFV infection and the antibody level *in vivo* of recombinant PRRSV live vector virus expressing the ASFV antigen protein.

**Table 4:** Tests of clinical samples.

Samples	Developed ELISA	Commercial ELISA
Positive	54	53
Negative	108	109

## 4 Discussion

Since 2018, the outbreak and prevalence of ASFV in China caused nearly 100% mortality (27). Subsequently, in 2020, less virulent genotype II ASFVs emerged in China due to natural mutations in the genomes of highly virulent viruses (9). These natural mutants showed reduced virulence and high transmissibility, causing chronic and persistent infections in pigs; but these pathogens were continuously shed *via* the oral and rectal routes at a low level, leading to difficulties and challenges for early diagnosis and control of ASF in China. Using OIE-recommended quantitative PCR and ELISA methods, researchers can accurately judge whether pigs are infected with wild-type ASFV. Recently, multiplex real-time qPCR was developed to provide a diagnostic tool for the differential detection of *B646L*, *I177L*, *MGF505-2R* and *EP402R* genes (28). For the early diagnosis and efficient prevention of circulating ASFV, antigen detection is very limited because of the marked decline in viral copy number. Currently, antibody detection of ASFV has become increasingly important (9). Antibody detection methods against p30, p54, or p72 of ASFV have been the most researched and applied in clinical diagnosis (29–31), and it is still necessary to explore more ASFV antigens.

The mammalian expression system can improve the expression and, more importantly, the recombinant protein obtained from this system can fold correctly to maintain its spatial conformation and protein activity. Contrastingly, the recombinant protein, produced using a prokaryotic expression system and structurally differing from the natural viral protein, primarily displays a linear epitope. Utilizing the suspension culture system can further increase the expression level due to the significantly increased cell density. Moreover, the suspension culture system yields relatively pure proteins owing to the serum-free culture medium and the simple purification process. Moreover, viral envelope proteins, difficult to be expressed in prokaryotic systems, perform better in eukaryotic systems. Here, we successfully expressed and purified pK205R in suspension-cultured 293i cells (**Figure 1**), which showed good immunogenicity in further experiments.

ASFV is a double-stranded DNA virus and the only DNA virus transmitted by insects. To date, there are no effective vaccines or drugs for the treatment of ASF. ASFV has huge particles and numerous encoded proteins (1, 17). At present, most of the research on protein function remains largely unknown, and a few of the existing studies on proteins have investigated the function of pK205R. pK205R can induce ER stress and consequently activate autophagy and the NF-κB signaling pathway (32). Early diffused expression of pK205R is closely related to viral particle production. pK205R has been identified as a strong antibody response and is largely expressed in the early stages of ASFV infection (19), suggesting that pK205R can be employed as a potentially useful serological diagnostic antigen. Herein, the indirect ELISA against pK205R revealed high sensitivity and good specificity, and showed great potential for ASF epidemic monitoring and control (**Table 4** and **Figure 7**). By the established ELISA method, 54 of 162 clinical serum samples tested positive, while 53 samples tested positive by the commercial kit, suggesting that the antibody detection against ASFV pK205R may be more sensitive than that against ASFV p30 in clinical serum samples. The mAb prepared from the recombinant pK205 vaccination showed specific reactivity to 293T cells transfected *K205R*-expression plasmid and recombinant PRRSV expressing pK205R (**Figures 2**, **5** and **6**), indicating the significance and effectiveness of our tool in research on pK205R function and live vector vaccines.

The epitopes of pK205R have rarely been reported, and merit further investigation as possible diagnostic tools. Our results showed that 2H11 specifically recognized the minimal linear epitope, ^2^VEPREQFFQDLLSAV^16^ (**Figure 3**). Further analysis showed that the sequences of this epitope were conserved among the 25 representative ASFV strains (**Figure 4**). So far, 24 ASFV genotypes have been identified based on the 3′-end sequence of the *B646L* gene, which encodes the major capsid protein p72 (33). In 2018, Georgia-07-like genotype II ASFV emerged in China and spread to other Asian countries (www.oie.int) (5). We selected multiple representative strains of different genotypes to analyze the conservation of pK205R and found that the epitope (^2^VEPREQFFQDLLSAV^16^) was 100% identical. A further prediction of the pK205R structure showed that the epitope was located at the N-terminus of pK205R and was exposed to the surface of the molecule, contributing to the understanding of the ASFV protein.

Currently, ASF is epidemic in China, and causes dramatic economic losses in pig industry. Although biological safety prevention and control measures can solve this disease epidemic in some extent. The costs have increased greatly which is not conducive to the healthy development of swine industry and will also bring a series of problems such as environmental pollution and rising pig prices. Safe and effective vaccines are urgently needed to prevent and control ASF. The biological characteristics of the pathogen of ASF—ASFV is complicated. Meanwhile, the ASFV invasion and induction of immune protection response have not been clearly addressed. Although many research teams have tried various technical methods, the safe and effective vaccine has not been innovated and developed. The reported vaccine candidates so far for ASF either have unsatisfactory immune efficacy or can cause significant biosafety issues by long-term use. Cellular immunity plays a vital role in the process of resisting ASFV infection. The advantage of live vector vaccine is that the antigen can be continuously expressed *in vivo*, and the vector virus itself can stimulate cellular immunity. In addition, the safety of live vector vaccine could be guaranteed. After expressing the antigen protein of ASFV, it can effectively stimulate ASFV-specific humoral immunity and cellular immunity, thus significantly improving the immune protection effect. Consider the perspective of biological safety and immune efficacy, live vector vaccine should be research direction of ASF vaccine in the future. At present, several viruses have been tried as live vectors to express ASFV antigen protein mainly including adenovirus, poxvirus, etc. Adenovirus is a replication-defective virus, while poxvirus has a large genome with many expressed products, and the foreign proteins expressed by them are difficult to stimulate a strong immune response. In this study, the foreign gene of ASFV K205R was inserted between ORF1b and ORF2 in the genome of PRRSV, based on the attenuated vaccine strain vHuN4-F112. ASFV K205R was translated as a new subgenomic mRNA under the control of the transcriptional regulatory sequence (**Figure 5**). Therefore, the expression of this foreign gene gradually increases along with the propagation of PRRSV. The N protein of PRRSV can induce the piglets to produce the largest amounts of antibodies detected by the IDEXX PRRSV Kit. The tendency of ASFV pK205R-induced antibody should be consistent with PRRSV N protein. But the level is thought to be lower than the antibody against N protein (**Figure 7**). Compared with other live vectors, PRRSV was a brand-new live vector, the recombinant PRRSV expressing ASFV pK205R was an important component of the recombinant PRRSV vaccine candidate strain against ASFV. It has also been proved that the strong humoral immune response could be induced. As a relatively better vector, PRRSV would not be affected by maternal derived antibody, and it has a relatively long viremia period that can continuously express the inserted foreign genes.

In our previous research, we constructed the recombinant virus rPRRSV-E2 which expressed E2 protein of classical swine fever virus with attenuated PRRSV vaccine strain (vHuN4-F112) as live vector. rPRRSV-E2 inoculated pigs did not show any side effects or tissue damages. Single immunization can stimulate a high-level and long duration of immune response (34). It could provide complete immune protection for piglets to resist the challenge of CSFV and HP-PRRSV (25), and has not been interfered and influenced by PRRSV and CSFV maternal antibody (35). This is far better than the anti-CSFV immune effect produced by another recombinant viruses expressing E2 protein with pseudorabies virus, adenovirus and poxvirus as vectors (36–38). These results indicate that, as a single-stranded RNA virus, PRRSV attenuated vaccine strain can express foreign proteins in pigs and induce strong immune response. PRRSV is an efficient live virus vector for pig vaccines. Especially, PRRSV and ASFV also share a common target cell—PAMs. Can the vaccine of ASF be developed by expressing the specific antigen protein of ASFV with PRRSV attenuated vaccine strain as a vector? Will can it effectively resist the infection of ASFV? Some studies have shown that the pK205R of ASFV has good immunogenicity and is a good antigen candidate for early diagnosis (21). In this study, pK205R of ASFV was selected as the foreign target antigen and was inserted into the live vector of PRRSV. The recombinant virus was successfully constructed and rescued, and the immunogenicity of the recombinant virus was verified. The mAb established in this study can be a useful tool to evaluate the expression level of pK205R in the recombinant virus (**Figures 5**, **6**), and the indirect ELISA method has great potential for the specific and effective detection of the antibody level *in vivo* of the recombinant virus (**Figure 6**), making them meaningful for future vaccine research on ASF.

Conclusively, recombinant ASFV pK205R was successfully expressed and purified from 293i suspension-cultured cells. The mAb 2H11 specifically recognized transiently expressed pK205R in MARC-145 cells, as well as specifically recognized recombinant PRRSV-infected cells expressing ASFVpK205R. These results indicate that the anti-pK205R mAb generated in this study has strong specificity and potential to be used in both basic and applied research on ASFV. Furthermore, the indirect ELISA method against pK205R, exhibiting good specificity and sensitivity, can potentially detect clinical ASFV infection and the antibody level of recombinant PRRSV live vector virus expressing ASFV antigen protein *in vivo*.

## Data availability statement

The raw data supporting the conclusions of this article will be made available by the authors, without undue reservation.

## Ethics statement

The animal study was reviewed and approved by The Ethics Committee of Shanghai Veterinary Research Institute, Chinese Academy of Agricultural Sciences (SV-20210723-Y02).

## Author contributions

This study was conceived and designed by FG and LL. All authors participated in the experiments. LL and SQ performed the experiments and prepared the figures. GL, JL and WT wrote the main manuscript text. SD and YZ analyzed the data. CL, YJ, and ZG prepared the manuscript. HZ, RZ, and GT made constructive comments on the experiments. All authors contributed to the article and approved the submitted version.
